# A study on moment tensor inversion of acoustic emission response on damaging localization of gas-bearing coal under load

**DOI:** 10.1038/s41598-022-20603-y

**Published:** 2022-09-30

**Authors:** Yue Niu, Enyuan Wang, Zhonghui Li

**Affiliations:** 1 State Key Laboratory for GeoMechanics and Deep Underground Engineering, China University of Mining and Technology, Xuzhou, 221116 China; 2 Key Laboratory of Coal Methane and Fire Control, Ministry of Education, China University of Mining and Technology, No. 1 Daxue Road, Tongshan District, Xuzhou, 221116 Jiangsu People’s Republic of China; 3 School of Mechanics and Civil Engineering, China University of Mining and Technology, Xuzhou, 221116 China; 4 School of Safety Engineering, China University of Mining and Technology, Xuzhou, 221116 China

**Keywords:** Environmental monitoring, Physics

## Abstract

During the deformation and fracture process, the acoustic emission (AE) signals can be produced for the of coal, rock and other solid materials, which revealing the damage localization evolution process. The effect of gas adsorption and pressure can change mechanical properties of coal mass and affect its damage development. Based on this, the experimental system for gas-bearing coal loading and AE monitoring was constructed, to analyze AE response characteristics under the joint action of loading stress and gas pressure on coal specimen. Afterwards, the damage localization evolution process of coal mass was studied with the moment tensor inversion method. Results showed that temporal response of AE signals was closely related to the damage degree and loading level of coal specimen, which could reveal its local severe damage and final failure characteristics. The spatial distribution and spread trend of AE fracture events inside coal specimen could be calculated through the moment tensor inversion method. It was basically consistent with the results of crack expansion on the specimen surface. The zones, where fracture events occurred intensively, gathered and spread in a continuous trend, were conductive to forming the macrocrack belt macroscopically. It could be regarded as the hazard zone with dynamic failure occurrence. Moreover, when the coal specimen faced the critical failure, the precursor characteristics of AE response appeared with the shear fracture events dominated markedly. The study results provide a new research idea for revealing the damaging localization evolution process under the coupling effect of stress and gas and lay the application foundation.

## Introduction

Coal mass belongs to a complex macromolecular organic matter, widely utilized in power generation, supply heating, steel smelting, coal chemical synthesis, et al.^1^. Coal resource is difficult to be mined, which needs to be extracted from deep underground under the joint influences of in-situ stress, gas pressure and mining disturbances^2–4^. This will inevitably change the original stable structure of coal and rock strata and may induce dynamic disasters (such as coal and gas outburst and rock burst), which can lead to the serious threat of casualty accident^5–8^.

Under the loading stress, the damage and deformation of coal mass lead to the final dynamic failure, which is the essential reason for the dynamic disaster in mining engineering. The weaker non-chemical bonds will break during the coal rupture process^8–10^. Microcosmically, the internal microcracks initiate, expand, coalesce and gradually form a crack gathered zone from the weak structural plane under loading stress. It finally induces the structural failure of coal mass with significant localized development characteristics^11,12^. Hence, it is of important research significance and engineering application value to monitor the failure feather and identify the failure hazard zone in coal mass, for the prevention and control of coal and rock dynamic disasters in the field^13^.

During damage, the fracture and crack propagation inside coal mass can increase the acoustic emission (AE) signals^14^. AE is an accompanying phenomenon that strain energy is released to the outside in the form of elastic waves during plastic deformation or fracture of coal mass. The sources of AE activities include plastic deformation, crack expansion and other material degradation^15–17^. The temporal signals consist of AE count, AE energy and amplitude, which can reveal damage evolution of coal mass. It is a relatively mature method and has been widely utilized to monitor materials, such as concrete, rock and coal. Liu et al.^18^ established an AE-based damage model of different bedding loaded coal. And the verification results show that the calculated theoretical stress–strain curves were basically consistent with the experimental ones. Wang et al.^19^ studied the relationship between AE and damage variable during fracture and evaluated damage and stress states of sandstone specimens based on AE parameters. Arash Behnia^15^ proposed the key role of artificial intelligence methods towards AE damage mode application. Meanwhile, if the AE event is regarded as a miniature earthquake event in space, AE parameters can be processed by the moment tensor inversion algorithm to obtain the spatial location of fracture events revealed by AE^20,21^. Further, the fracture types and the expand trend also can be analyzed consequently. Othsu^22^ programmed and applied the simplified *Green’s* function for the moment tensor inversion using AE signals. Liu et al.^23^ constructed a moment tensor inversion algorithm of AE with far-field P-wave and verified its correctness and reliability through an artificial experiment. Chai et al. established a method for studying fracture mechanisms and evolution process of intact rock and joint rock based on the moment tensor theory and *P–T* diagram, and then applied it to analyze the stability of underground mines^24^. Simultaneously, it cannot be ignored that the influence of gas adsorption with high-pressure and high-content on the coal mass underground^25^. When the gas adsorbs inside the coal mass, it can change the micropores and crack structures, and further affects mechanical properties and loading states of coal mass. Meanwhile, the damaging evolution and failure feather are changed due to the gas. Consequently, it is critical and necessary to reveal the damage development process of coal mass under the joint action of stress and gas^26^.

After the adequate consideration on the joint action of loading stress and gas pressure, the experimental system for gas-bearing coal loading and AE monitoring was established to analyze AE response characteristics. Further, the AE moment tensor inversion results were utilized to analyze the distribution and development of micro-fracture events. It is promising to provide a new idea for revealing the damaging localization process of coal mass under the coupling effect of stress and gas.

## Experimental scheme

### Experimental system

**Figure 1:**
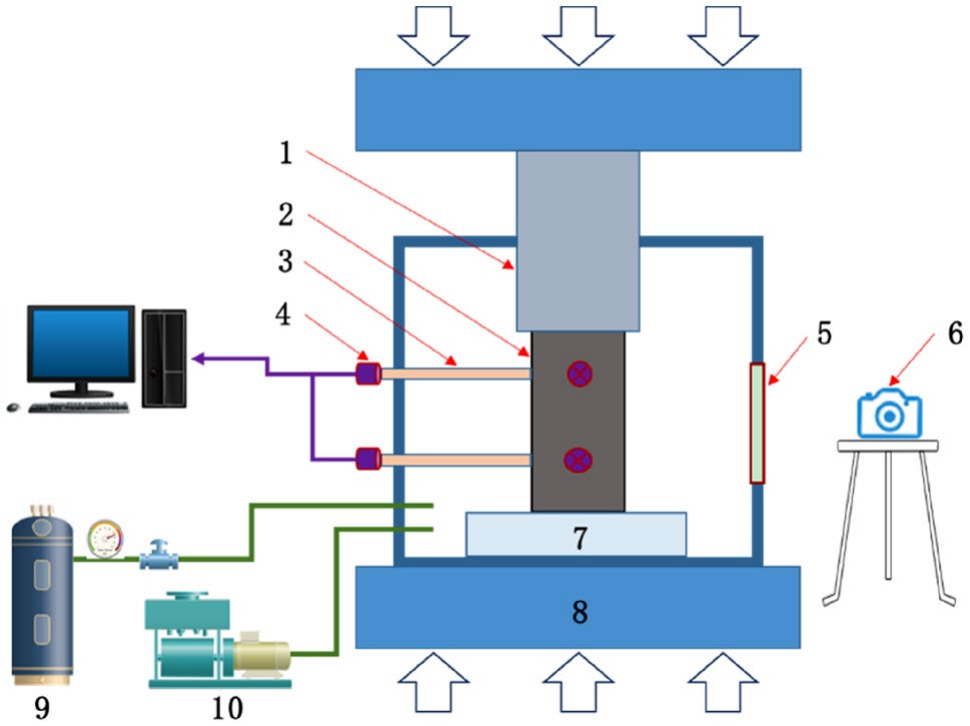
Diagrammatic sketch of experimental system for gas-bearing coal loading and AE monitoring. 1—Loading shaft; 2—coal specimen; 3—waveguide rod; 4—ae sensor; 5—visible window; 6—camera; 7—heel block for loading; 8—loading platform; 9—gas cylinder; 10—vacuum pump.

As shown in Fig. 1, an experimental system for gas-bearing coal loading and AE monitoring was established. and mainly included a visual sealed cylinder, an electro-hydraulic servo loading platform, an AE acquisition device, a high-speed camera and a gas charging and discharging device. It could provide a sealed environment for gas adsorption by coal and synchronously test the change of loading stress, strain and AE signals during damaging process of coal specimens under the joint action of loading stress and gas pressure. Moreover, the physical object images of crack expansion inside coal specimens also could be photographed by camera in real time.

#### Visual sealed cylinder

**Figure 2:**
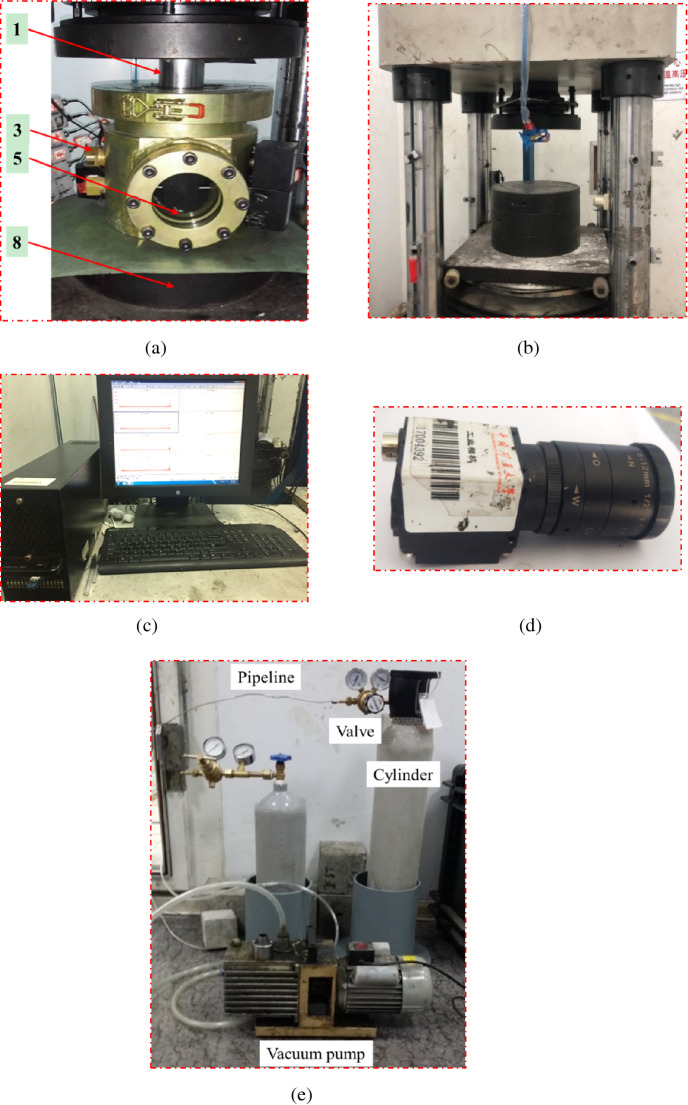
Physical object images of key parts of the experimental system. (**a**) Visible sealed cylinder. (**b**) Electro-hydraulic servo loading platform. (**c**) AE acquisition device. (**d**) High-speed camera. (**e**) Gas charging and discharging device.

The physical object of the visual sealed cylinder was shown in Fig. 2a. It can be seen that a visible window was processed on the front of the cylinder (by arranging tempered glass), through which the camera could photograph crack expansion on the specimen in real time. A loading shaft on the upper end of the cylinder could move up and down while ensuring sealing of the cylinder to apply loading stress on the specimen. The cylinder was well sealed and could withstand a maximum gas pressure of 5 MPa, which provided a simulated environment for coal to fully adsorb gas.

#### Electro-hydraulic servo loading platform

The physical object of the electro-hydraulic servo loading platform was shown in Fig. 2b. The platform imposed stress on the specimen through electro-hydraulic servo loading. The platform with the maximum load of 3000 kN could be programmed with different experimental schemes, realizing different loading modes, such as force control or displacement control, and recorded the changing curves of the stress and strain in real time.

#### AE acquisition device

The physical object of the AE acquisition device was demonstrated in Fig. 2c. This device is equipped with a multi-channel AE data acquisition system with a PCI-Express bus structure produced by Physical Acoustics Corporation in the United States. And it can collect AE signals during the deformation and fracture of specimen in real time, and synchronously analyze waveforms of signals and locate AE events in the three-dimensional (3D) space. The instrument has a bandwidth of 1 kHz–1.2 MHz and the maximum sampling frequency of 10 MHz.

It is worth noting that the conventional AE sensors cannot work in high-pressure gas^27^. In previous research, the typical approach is to place sensors on the surface of the cylinder containing high-pressure gas, and use the loading shaft and cylinder wall as transmission media to receive AE signals generated by the specimen in cylinder. Though this method, the signals collected are greatly disturbed, and it is difficult to spatially locate fracture events through the time difference of various sensor’s signals. Therefore, the waveguide rod was designed from Fig. 1 in this study, which passed through the cylinder wall to make AE sensors outside the cylinder indirectly contact with the specimen in cylinder. So it could accurately monitor the AE signals of coal mass and allow location and calculation. The waveguide rod and cylinder wall were connected by O-type sealing rings at the same time to ensure the airtightness of the cylinder. Both ends of the waveguide rod were coated with vaseline as a coupling agent to enhance transmission effect of AE signals.

#### High-speed camera

The crack expansion process of the specimen was photographed with a USB2.0MV-UB500 high-definition industrial camera at a high shooting speed. The physical object of the industrial camera was shown in Fig. 2d. The camera has a maximum shooting frequency of 50 Hz and a corresponding optical resolution of 2592 × 1944 pixels with a pixel size of 2.2 μm × 2.2 μm. The camera and its supporting measurement software could realize real-time acquisition of specimen images and accurate measurement of crack expansion.

#### Gas charging and discharging device

As shown in Fig. 2e, the original gas in the cylinder was pumped out by a vacuum pump, and then the cylinder was filled with gas to a certain pressure from a gas cylinder. Note that the ultimate pressure of the vacuum pump was 100 Pa.

### Experimental scheme

**Figure 3:**
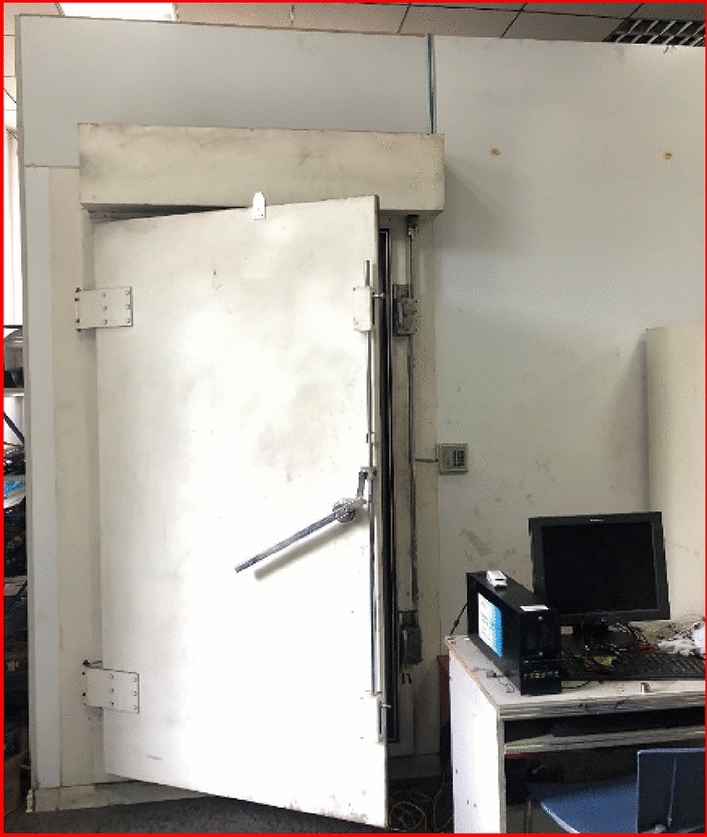
Physical object images of the electromagnetic shielding enclosure.

**Figure 4:**
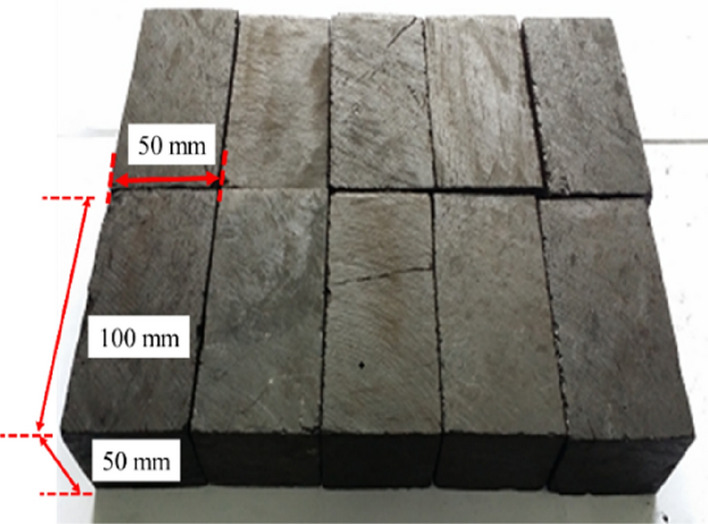
Physical object images and dimension of the coal specimens.

**Figure 5:**
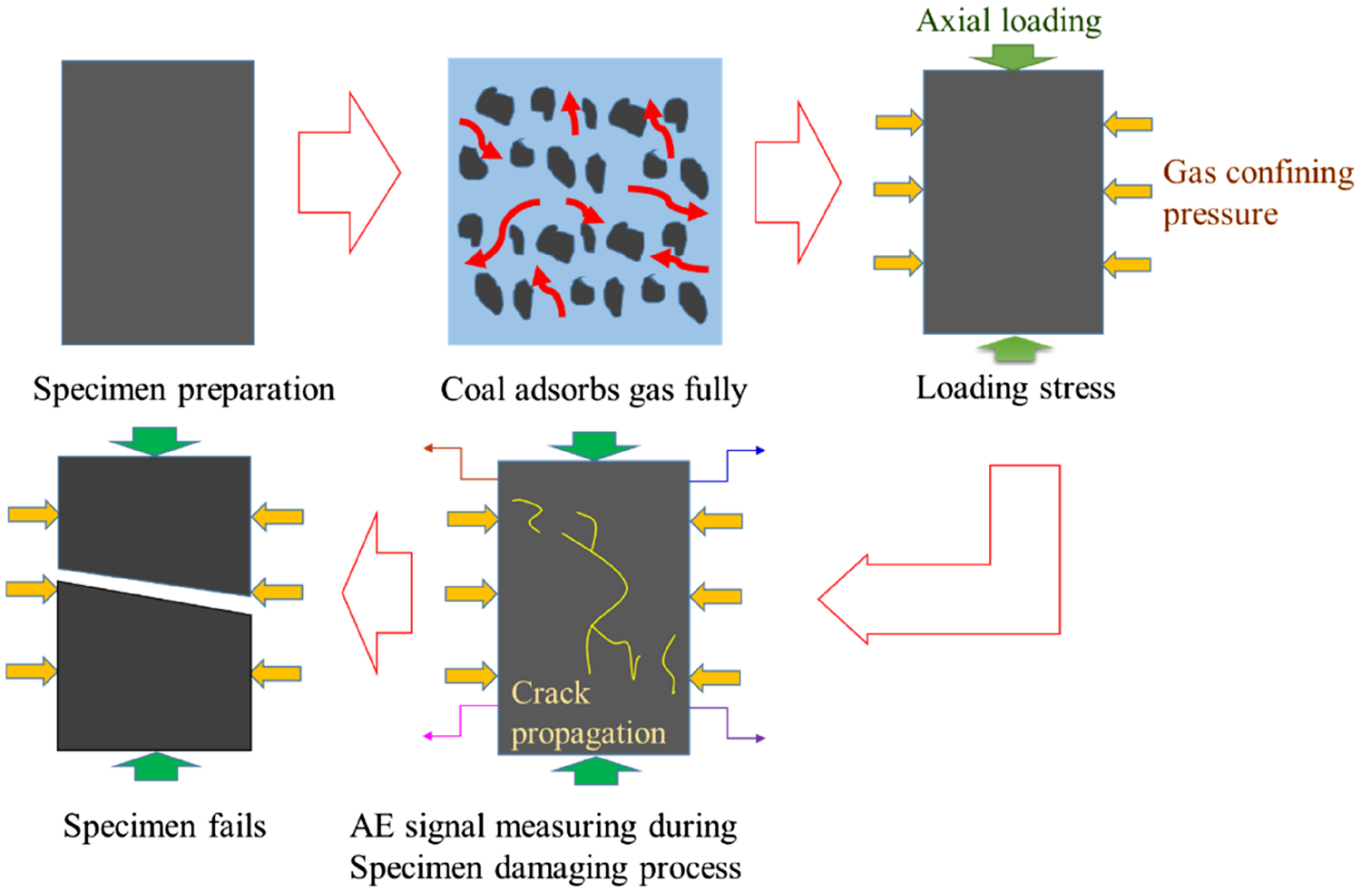
Flow chart of experimental process under load until the gas-bearing coal failure.

As shown in Fig. 3, the experiment of AE monitoring in failure of gas-bearing coal under load was conducted in an electromagnetic shielding enclosure (located in China University of Mining and Technology). The shielding enclosure could effectively isolate the interference of the environmental noise and operation of mechanical and electrical equipment to signal acquisition. The specimen was taken from No. 5 coal seam of Yangzhuang Coal Mine, Anhui Province, China, and was processed into rectangles with dimensions of 50 mm × 50 mm × 100 mm, as shown in Fig. 4. After sample preparation, the experiment was conducted according to the following steps and the flow chart of experimental process was shown in Fig. 5.

① The airtightness of the sealed cylinder was checked to ensure good sealing effects of the cylinder. The various parts of the experimental system were connected and the insulation paper was placed on the upper and lower parts of the cylinder.
② The coal specimen was placed into the cylinder and the waveguide rod passed through the cylinder wall. The rod was in contact with the specimen in the cylinder with AE sensors outside the cylinder. And vaseline as the coupling agent was smeared at connections of AE sensor and specimen.
③ The original gas in the cylinder was discharged and then the cylinder was sealed. The cylinder was vacuumized by using the vacuum pump, and then was filled with gas. After reaching the pre-set pressure value (2 MPa), the pressure in the cylinder was kept stable, so that coal specimen could fully adsorb gas.
④ When the process of gas adsorption by coal specimen reached the state of dynamic equilibrium, all mechanical and electrical equipments were turned on and the experimental parameters were set. The specimen was uniaxially compressed at the velocity of 25 N/s and the signals including stress, strain and AE were synchronously collected. Moreover, the crack expansion in the specimen was photographed in real time.
⑤ After main fracture occurred in the specimen, the experiment had come to an end and the gas in the cylinder was pumped out.

## Moment tensor inversion method of AE signals

### Theory analysis of moment tensor inversion using AE signals

Under the joint action of loading stress and gas pressure, the plastic deformation and fracture behaviors occur inside gas-bearing coal, while AE signals are generated^14^. The micro-fracture occurrence or displacement change at fracture locations caused by crack expansion is the essence of AE generation. If the fracture source is regarded as a particle, the wave equation of the particle displacement during the generation of fracture events can be transformed into the convolution of moment tensor and *Green’s* function. Through moment tensor inversion, micro-fracture mechanisms can be known and the eigenvalues and eigenvectors are calculated. Therefore, the spatial location of fracture events as well as spatial orientation and expansion trend of the fracture surface can be obtained.

If the change of displacement of the particle with time is considered as a continuous elastic vibration process, the vibration laws will be suitable for elastic wave propagation criteria, thus the following formula is obtaining^28,29^.

1 $$ u_{k} \left( {x,t} \right) = \int_{ - \infty }^{ + \infty } {\int_{S} {G_{ki} \left( {x,t,r,t^{\prime}} \right)f_{i} \left( {r,t^{\prime}} \right)} } dSdt^{\prime} $$

where, $u_{k} \left( {x,t} \right)$, $G_{ki} \left( {x,t,r,t^{\prime}} \right)$ and *S* represent the displacement of a fracture point at position *x* at the moment *t*, *Green’s* function and range of the integrated fracture surface, respectively.

When only the far-field term of the displacement field of *P*-wave data in AE signals is considered, the relationship between the radial displacement and the moment tensor at the fracture point can be obtained as follows.

2 $$ u_{k} \left( {x,t} \right) = \frac{{\gamma_{i} \gamma_{j} \gamma_{k} }}{{4\pi \rho_{c} v_{p}^{3} r_{d} }}\dot{M}_{i,j} \left( {t - \frac{{r_{d} }}{{v_{p} }}} \right) $$

where, $\gamma_{i}$, $\gamma_{j}$ and $\gamma_{k}$ indicate the components of each coordinate axis corresponding to the vibration wave vector from the fracture point to the measuring point; $r_{d}$, $v_{p}$, $\rho_{c}$ and $\dot{M}_{ij}$ denote the distance from the fracture point to the measuring point, *P*-wave velocity, specimen density and derivative of the moment tensor to time, respectively.

Assuming that the source is synchronous, then the temporal and spatial relationships of the moment tensor can be separated as follows^30^.

3 $$ p^{\left( i \right)} u^{\left( i \right)} = k_{p}^{\left( i \right)} \left[ {\begin{array}{*{20}c} {\gamma_{1}^{\left( i \right)} \gamma_{1}^{\left( i \right)} \gamma_{1}^{\left( i \right)} } \\ {2\gamma_{1}^{\left( i \right)} \gamma_{1}^{\left( i \right)} \gamma_{2}^{\left( i \right)} } \\ {2\gamma_{1}^{\left( i \right)} \gamma_{1}^{\left( i \right)} \gamma_{3}^{\left( i \right)} } \\ {\begin{array}{*{20}c} {\gamma_{1}^{\left( i \right)} \gamma_{2}^{\left( i \right)} \gamma_{2}^{\left( i \right)} } \\ {2\gamma_{1}^{\left( i \right)} \gamma_{2}^{\left( i \right)} \gamma_{3}^{\left( i \right)} } \\ {2\gamma_{1}^{\left( i \right)} \gamma_{3}^{\left( i \right)} \gamma_{3}^{\left( i \right)} } \\ \end{array} } \\ \end{array} } \right]^{{T}} \cdot \left[ {\begin{array}{*{20}c} {\begin{array}{*{20}c} {M_{11} } \\ {M_{12} } \\ {M_{13} } \\ \end{array} } \\ {M_{22} } \\ {M_{23} } \\ {M_{33} } \\ \end{array} } \right] $$

where, $p^{\left( i \right)}$ and $u^{\left( i \right)}$ represent the direction and amplitude of the initial fluctuation of the *i*_th_ measuring point, respectively; $k_{p}^{\left( i \right)} {\text{ = exp}}\left( { - \frac{\pi f}{{v_{p} Q_{p} }}r_{d}^{\left( i \right)} } \right)\frac{1}{{{4}\pi \rho_{c} v_{p}^{3} r_{d}^{\left( i \right)} }}$; $r_{d}^{\left( i \right)}$ and $\gamma_{l}^{\left( i \right)}$ indicate the distance from the fracture point to the *i*th measuring point and component of the coordinate axis *l* corresponding to the *i*th measuring point, respectively.

where,

4 $$ p^{\left( i \right)} = \left\{ {\begin{array}{ll} {1}, & \quad {\text{if receiving point is above fracture point and initial waveform moves up}} \\  {- 1}, & \quad {\text{if receiving point is above fracture point and initial waveform moves down}} \\ {1}, & \quad {\text{if receiving point is upon fracture point and initial waveform moves down}} \\  {- 1}, & \quad{\text{if receiving point is upon fracture point and initial waveform moves up}} \\ \end{array} } \right. $$

The moment tensor ${\varvec{M}}_{ij}$ of AE meets the momentum conservation, only including six independent components, namely $M_{ij} = M_{ji}$. Assuming that three eigenvalues are $M_{1}$, $M_{2}$ and $M_{3}$ ($M_{1} \ge M_{2} \ge M_{3}$), and the eigenvectors corresponding to the eigenvalues are ${\varvec{e}}_{1}$, ${\varvec{e}}_{2}$ and ${\varvec{e}}_{3}$. A certain moment tensor can be decomposed into three parts including isotropic (*ISO*), pure double couple (*DC*) and compensated linear vector dipole (*CLVD*). The ISO part is represented by a matrix of three equal eigenvalues, and only shows the volumetric strain. The DC part indicates the linear vector dipole combination of pure shear failure. And the *CLVD* implies the remaining part in the partial tensor expect for the *DC* part. In the principal coordinate system (${\varvec{e}}_{1}$, ${\varvec{e}}_{2}$, ${\varvec{e}}_{3}$), the moment tensor can be diagonalized and decomposed as follows^31^.

5 $$ \begin{aligned} {\varvec{M}}_{{{\varvec{ij}}}} & = \left[ {\begin{array}{*{20}c} {M_{11} } & {M_{12} } & {M_{13} } \\ {M_{21} } & {M_{22} } & {M_{23} } \\ {M_{31} } & {M_{32} } & {M_{33} } \\ \end{array} } \right] = \left[ {\begin{array}{*{20}c} {M_{1} } & 0 & 0 \\ 0 & {M_{2} } & 0 \\ 0 & 0 & {M_{3} } \\ \end{array} } \right] = {\varvec{M}}^{ISO} + {\varvec{M}}^{DC} + {\varvec{M}}^{CLVD} \\ & = \frac{1}{3}\left[ {\begin{array}{*{20}c} {tr\left( M \right)} & 0 & 0 \\ 0 & {tr\left( M \right)} & 0 \\ 0 & 0 &{tr\left( M \right)} \\ \end{array} } \right] \\ & \quad + \frac{1}{2}\left( {M_{1} - M_{3} } \right)\left[ {\begin{array}{*{20}c} 1 & 0 & 0 \\ 0 & 0 & 0 \\ 0 & 0 & { - 1} \\ \end{array} } \right] \\ & \quad + \frac{1}{6}\left( {2M_{2} - M_{3} - M_{1} } \right)\left[ {\begin{array}{*{20}c} { - 1} & 0 & 0 \\ 0 & 2 & 0 \\ 0 & 0 & { - 1} \\ \end{array} } \right] \\ \end{aligned} $$

where, $tr\left( M \right)$ represents the sum of all eigenvalues of the moment tensor ${\varvec{M}}_{ij}$; ${\varvec{M}}^{ISO}$, ${\varvec{M}}^{DC}$ and ${\varvec{M}}^{CLVD}$ denote the components at the three corresponding positions in the above equation, respectively.

### Judgment of fracture types of events and motion vectors

If the proportions of the above three parts in the moment tensor are defined as $P^{ISO}$, $P^{DC}$ and $P^{CLVD}$, then the type of fracture events can be judged according to the proportion values. The judgment basis is shown as follows.

6 $$ \left. {\begin{array}{ll} {P^{DC} \ge 60\%} , & \quad {\text{ shear failure}} \\ {P^{ISO} \ge 60\%} ,& \quad {\text{ tensile failure}} \\ {P^{DC} < 60\%} \; {\text{and }}P^{ISO} < 60\%, & \quad  {\text{ mixed failure}} \\ \end{array} } \right\} $$

The intensity of fracture events can be measured by the seismic moment $M_{0}$, which can be calculated as follows^32^.

7 $$ M_{0} { = }\sqrt {\sum\limits_{i = 1}^{3} {\left( {\frac{1}{2}M_{j}^{2} } \right)} /2} $$

The eigenvectors corresponding to the eigenvalues $M_{1}$, $M_{2}$ and $M_{3}$ of the moment tensor are assumed as ${\varvec{e}}_{1}$, ${\varvec{e}}_{2}$ and ${\varvec{e}}_{3}$. Supposing that the cracks can be approximately regarded as a plane and the normal vector and motion vector separately are ${\varvec{n}}_{{\varvec{e}}}$ and ${\varvec{l}}_{{\varvec{e}}}$, then the eigenvectors can be calculated as follows.

8 $$ \left. {\begin{array}{*{20}c} {{\varvec{e}}_{1} = {\varvec{l}}_{{\varvec{e}}} { + }{\varvec{n}}_{{\varvec{e}}} } \\ {{\varvec{e}}_{2} = {\varvec{l}}_{{\varvec{e}}} { \times }{\varvec{n}}_{{\varvec{e}}} } \\ {{\varvec{e}}_{3} = {\varvec{l}}_{{\varvec{e}}} { - }{\varvec{n}}_{{\varvec{e}}} } \\ \end{array} } \right\} $$

**Figure 6:**
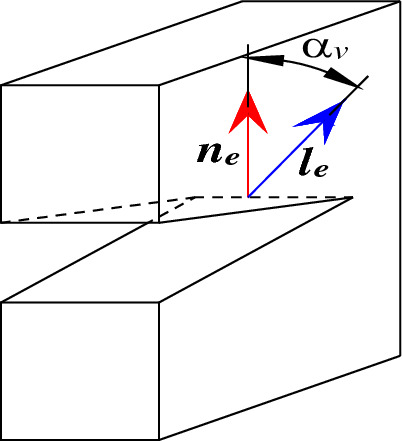
Schematic diagram of the angle for characterizing fracture modes.

By calculating ${\varvec{n}}_{{\varvec{e}}}$ and ${\varvec{l}}_{{\varvec{e}}}$ according to ${\varvec{e}}_{1}$, ${\varvec{e}}_{2}$ and ${\varvec{e}}_{3}$, the motion vector of fracture events can be obtained. As displayed in Fig. 6 and Formula (9), $\alpha_{v}$ is defined as the angle characterizing fracture mode.

9 $$ \alpha_{v} = \left| {\frac{{{\varvec{l}}_{{\varvec{e}}} \cdot {\varvec{n}}_{{\varvec{e}}} }}{{\left| {{\varvec{l}}_{{\varvec{e}}} } \right|\left| {{\varvec{n}}_{{\varvec{e}}} } \right|}}} \right| $$

In theory, if the fracture event belong to pure tensile fracture, the motion vector of the fracture surface is parallel to the normal vector, so $\alpha_{v} = 0^\circ$. Meanwhile, if the events are judged as pure shear fracture, the motion direction of the fracture surface is perpendicular to the normal vector, so $\alpha_{v} = 90^\circ$. In the actual calculation, it is difficult to simply define a fracture event as pure tensile fracture or pure shear fracture, and the fracture of the event is still mainly judged according to Formula (6). On this basis, if $\alpha_{v}$ is closer to 90°, the shear fracture has a higher proportion. Instead, if $\alpha_{v}$ is closer to 0°, the proportion of tensile fracture is higher.

### Accurate location of fracture events

Various AE measuring points are arranged on the surface of gas-bearing coal. So according to the arrival time of signals received at each measuring point after the fracture event occurs, the fracture point can be located, as follows.

10 $$ r_{d} = \sqrt {\left( {x_{0} - x_{i} } \right)^{2} + \left( {y_{0} - y_{i} } \right)^{2} + \left( {z_{0} - z_{i} } \right)^{2} } = v_{p} \left( {t_{i} - t} \right) $$

where, $\left( {x_{0} ,y_{0} ,z_{0} } \right)$ and $\left( {x_{i} ,y_{i} ,z_{i} } \right)$ represent the coordinates of the fracture point and the *i*_th_ measuring point, respectively; *t* and *t*_*i*_ indicate the moment when the fracture event occurs and the onset time of event waveforms received by the *i*th measuring point, respectively.

As coal mass are actually heterogeneous, the above calculation results will inevitably have errors. In existing studies, the absolute value of the difference between the onset time of actual waveforms at each measuring point and the calculated arrival time is taken as an objective function^23^. The point at which the minimum value is obtained by solving the objective function is the optimal solution to errors as follows.

11 $$ y = \frac{1}{{n_{s} }}\sum\limits_{i = 1}^{{n_{s} }} {\left| {\left( {t_{i} - t} \right) - \frac{1}{{v_{p} }}\sqrt {\left( {x_{0} - x_{i} } \right)^{2} + \left( {y_{0} - y_{i} } \right)^{2} + \left( {z_{0} - z_{i} } \right)^{2} } } \right|} $$

Due to the presence of the absolute value, the objective function calculated by Formula (11) is not derivable everywhere. The simplex algorithm can be used for indirect calculation^19^. The calculation steps are shown as follows.

**Figure 7:**
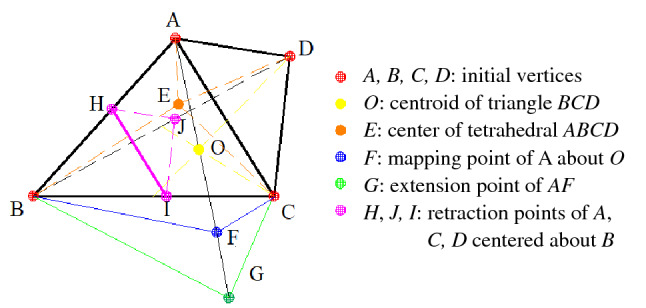
Schematic diagram of tetrahedral deformation based on simplex algorithm.
② The centroid point *O* of a graph composed of vertices except for the worst point *A* is calculated. Then the mapping point *F* of point A to point *O* is obtained through basic deformation operation. Along the *AF* direction, distance from point *F* to point *G* can be extended proportionally. In this case, the segment length is *AF* = *FG*. *E* is the center of the tetrahedron *ABCD*. The objective function values of each point are calculated.
③ Based on Formula (12), the objective functions including *y*_*A*_, *y*_*B*_, *y*_*E*_, *y*_*G*_ and *y*_*F*_ are compared and the corresponding vertices are replaced. If none of the conditions in Formula (12) is met, then the tetrahedron is retracted proportionally with *B* as the center (that is, points *A*, *C* and *D* are separately replaced by points *H*, *I* and *J*, and the triangle *ACD* is similar and parallel to the triangle *HIJ*).
  12 $$ \left\{ \begin{aligned} & y_{F} \le y_{B} {\text{ and }}y_{G} \ge y_{B}, \;\;{\text{ then }}A{\text{ is replaced by }}F \, \hfill \\ & y_{F} \le y_{B} {\text{ and }}y_{G} \le y_{B} , \;\;{\text{ then }}A{\text{ is replaced by }}G \hfill \\ & y_{F} > y_{B} {\text{ and }}y_{F} \le y_{A}, \;\; {\text{ then }}A{\text{ is replaced by }}F \hfill \\ & y_{F} > y_{B} {, }y_{G} \ge y_{B} , \;\;\;\;\;\;{\text{ and }}y_{E} \le y_{A} {\text{ then }}A{\text{ is replaced by }}E \hfill \\ \end{aligned} \right. $$
④ After updating the vertices through the above steps, the tetrahedron is reconstructed. The above steps are repeated until the stopping criterion for iteration is satisfied. That is, the objective function value meets the pre-set error allowable range. In this case, the vertex with the lowest value of the objective function in the tetrahedron is the location of the fracture point finally calculated.

① As shown in Fig. 7, four measuring points preliminarily calculated to be closest to the fracture point are selected as the vertices (*A*, *B*, *C* and *D*) of a tetrahedron of the initial iterative structure. Meanwhile, the above four vertices as fracture points are substituted into the objective function for calculation. It is assumed that the objective function is maximized (*y*_*worst*_) at point *A*, namely the worst point, and the minimum value *y*_*best*_ of the objective function is obtained at point *B*, namely the best point.

### Identification of onset and initial amplitude of waveforms

After the fracture event occurs, the onset time and initial amplitude of P-wave waveforms can be accurately identified, and the following steps are followed.

1. Determination of a threshold of background noise
  The background noise ${\dot{\text{A}}}$ at one end is selected and the temporal sequence is $\left\{ {a_{i} } \right\}$. By calculating the average $m_{A}$ and standard deviation $\sigma_{A}$ of the sequence, the signal threshold $T_{A}$ of background noise is expressed as follows.
  13 $$ T_{A} = m_{A} \pm 4\sigma_{A} $$
2. Determination of trigger time of waves
  The point *L*_*t*_ where the amplitude exceeds the noise threshold *T*_*A*_ for the first time in the waveform data is recorded. By taking this point as the objective point, *L*_*f*_ sampling points forward and *L*_*b*_ sampling points backward are selected, which ensure that the waveform segment *R* in the time window [*L*_*t*_ − *L*_*b*_, *L*_*t*_ + *L*_*f*_] contains part of signals of pure background noise and the trigger time information of P-waves. The formula for calculating the *Akaike Information Criterion* ’s function (*AIC*) is defined as follows^33^.
  14 $$ AIC\left( w \right){ = }w\log_{10} \left\{ {{\text{var}} \left[ {R\left( {1,w} \right)} \right]} \right\} + \left( {L_{r} - w - 1} \right)\log_{10} \left\{ {{\text{var}} \left[ {R\left( {w + 1,L_{r} } \right)} \right]} \right\} $$
  where, *L*_*r*_ indicates the length of segment *R* (the number of sampling points contained) and $L_{r} { = }L_{b} { + }L_{f} { + }1$; *w* represents the index number of segment *R* (serial number of sampling points in this segment) and values in the range of $\left[ {1,L_{r} } \right]$; ${\text{var}}$ denotes the variance function (var = σ^2^) of the specimen.
  If the *AIC’s* function has the minimum value at *w*_min_, the potential point of trigger time of waves will be $w_{\min } + L_{t} - L_{b} - 1$. Figure 8 shows a schematic example of the *AIC’s* function.

**Figure 8:**
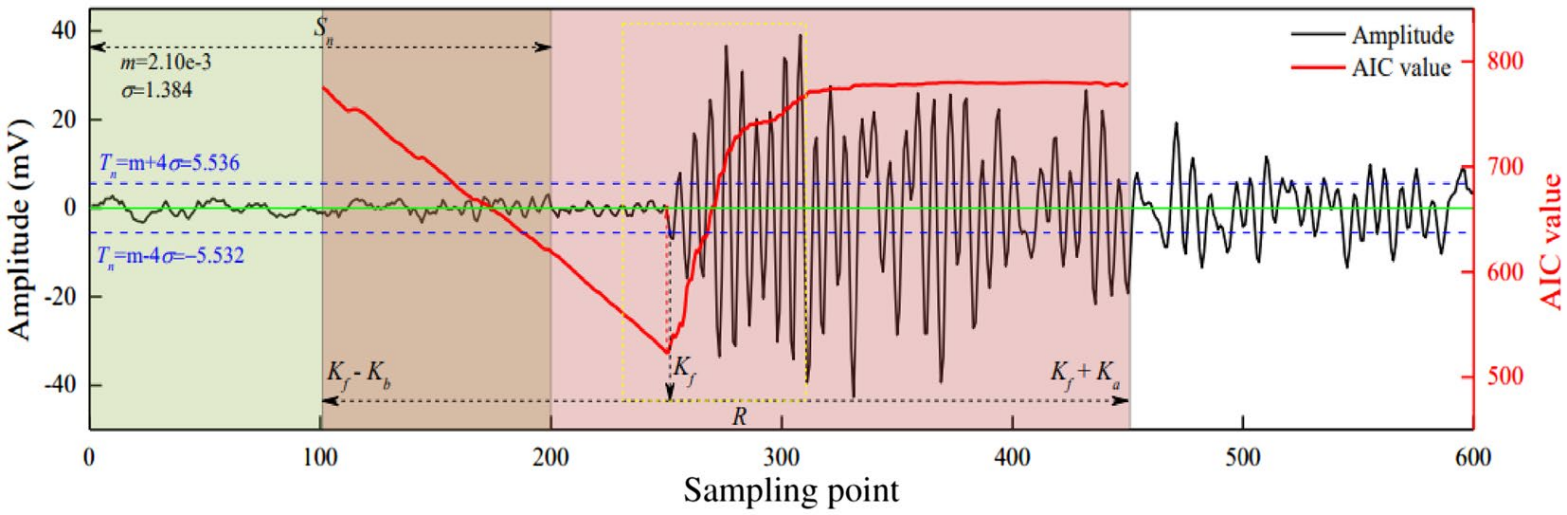
Calculation example of *AIC’s* function.
3. Verification of trigger time of waves
  If the potential point of trigger time is confirmed as the real point of trigger time, both conditions should be met ulteriorly as follows. ① In the next 30 points from this point, the waveform amplitude exceeds the threshold *T*_*A*_ of the background noise at least four times, namely goes beyond the interval [− *T*_*A*_, *T*_*A*_]. ② In the next 50 points from this point, the signs of the waveform amplitude change at least five times. Otherwise, the data of the arrival time will be discarded.
  After determining the trigger time of waves, the initial amplitude and direction (positive and negative values) are determined. In other words, starting from *w*_min_, the amplitude *R*_*i*_ of the sampling point that can satisfy Formula (14) as early as possible is recognized as the initial amplitude *V*_*P*_. Figure 9 shows the identification examples of trigger time and amplitude of a same fracture event by six AE sensors.
  15 $$ \left\{ {\begin{array}{*{20}c} {{\text{When }}R_{{w_{\min } + 1}} > R_{{w_{\min } + 1}} ,{\text{then }}R_{i + 1} < R_{i} } \\ {{\text{When }}R_{{w_{\min } + 1}} < R_{{w_{\min } + 1}} ,{\text{then }}R_{i + 1} > R_{i} } \\ \end{array} } \right. $$

**Figure 9:**
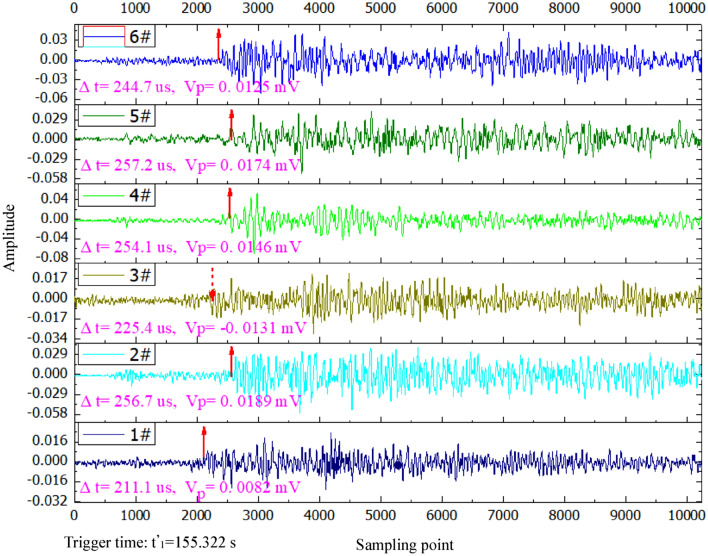
Identification examples of trigger time and amplitude of a same fracture event by six AE sensors.

## Experimental results

### Experimental results of temporal response of AE signals

#### Temporal variation of the axial stress

**Figure 10:**
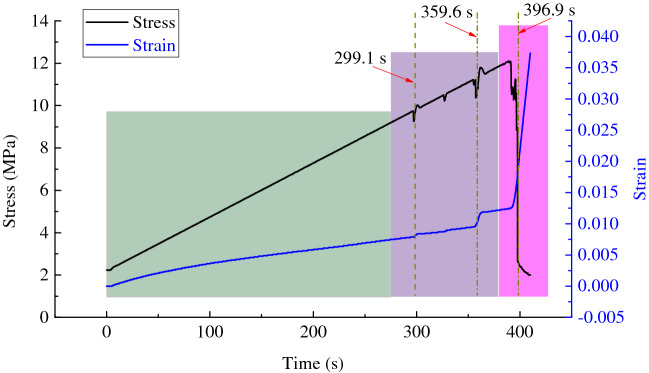
Experimental results of stress and strain during loading process of gas-bearing coal.

The changes of the axial stress and strain with the loading time during failure of gas-bearing coal under load were shown in Fig. 10. Due to the action of gas pressure, the initial axial stress of the specimen was not zero. As the loading continues, the damage of specimen was constantly intensified and the axial stress and strain tended to rise. Because of pre-existing cracks and pores in coal mass and thereby inhomogeneity of coal material, the specimen was not an ideal elastic–plastic body and many local fracture and serious damage would occur before the specimen failed. Both stress and strain change slightly at 299.1 and 359.6 s, which indicates that serious damage appeared in the specimen at that moments and crack expansion started and intensified. After that, the stress and strain rose continuously. At 396.9 s, the specimen failed and the stress changed violently and decreased rapidly. Meanwhile, the strain increased rapidly.

#### Temporal variation of AE

AE count reflects the occurrence frequency of micro-fractures during the damaging process of coal specimen, while AE energy shows the magnitude of released energy induced by the micro-fracture. Both indexes of AE signals can be used to reflect the damage evolution and energy release of coal specimen and further monitor and forecast its failure.

**Figure 11:**
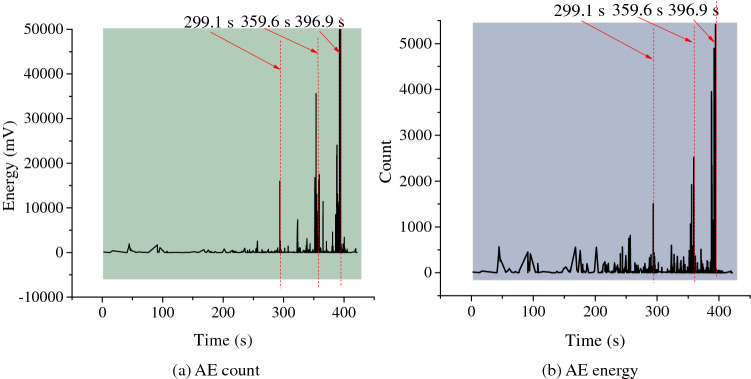
Experimental results of AE indexes during loading process of gas-bearing coal. (**a**) AE count. (**b**) AE energy.

The change of AE signals with the loading time in failure of gas-bearing coal was shown in Fig. 11. In the early loading stage, there were few AE signals. However, as the loading continued, the stress constantly increased, and AE count and energy tended to rise. At 299.1 and 359.6 s, the AE count and energy suddenly rose in a pulse pattern, with a high amplitude. It reflected the sharp increase of micro-fracture signals in coal mass quickly, which released a large amount of elastic energy. And it is corresponded to the moments of sudden change of the stress and strain in Fig. 10. Hence, it could be regarded as interactive supplementary evidence to further verify that serious damage had occurred in the specimen at the corresponding moments. At 396.9 s, the AE count and energy met the maximum values, indicating that the fracture scale quickly increased due to rapid expansion and coalescence of microcracks abundant in coal mass. As a result, the final failure of specimen occurred, and a large number of elastic energy was released. After that, AE signals reduced rapidly and then almost disappeared.

The above research shows that the dominant mechanism of dynamic failure for gas-bearing coal is fracture caused by the rapid crack expansion and coalescence in coal mass under the joint action of stress and gas. Moreover, elastic energy is released outside and AE signals are generated. Coal mass shows mechanical behaviors, such as compaction, closure, initiation and expansion of crack during loading process, which is the main cause for temporal response of AE signals.

### Location results based on moment tensor inversion using AE signals

**Figure 12:**
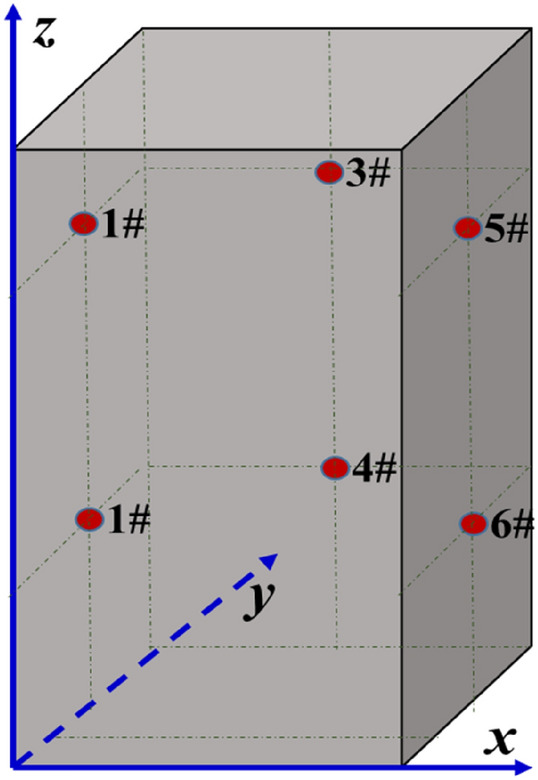
Schematic diagram of AE monitoring points layout for moment tensor inversion.

As shown in Fig. 12, a total of six AE monitoring points were arranged on the axes of left, rear and right views of the specimen in this experiment. The sensors were not arranged on front view, which allow photography of crack expansion of the sample from the outside of cylinder through the above visible window. The Cartesian Coordinate System was established with 1 mm as the unit. The coordinates of 1#–6# AE monitoring points were shown in Table 1.

**Table 1:** Coordinates of AE monitoring points for the moment tensor inversion.

Monitoring point	Coordinates (mm)			Monitoring point	Coordinates (mm)		
	*x*	*y*	*z*		*x*	*y*	*z*
1 #	0	25	75	4 #	25	50	25
2 #	0	25	25	5 #	50	25	75
3 #	25	50	75	6 #	50	25	25

**Figure 13:**
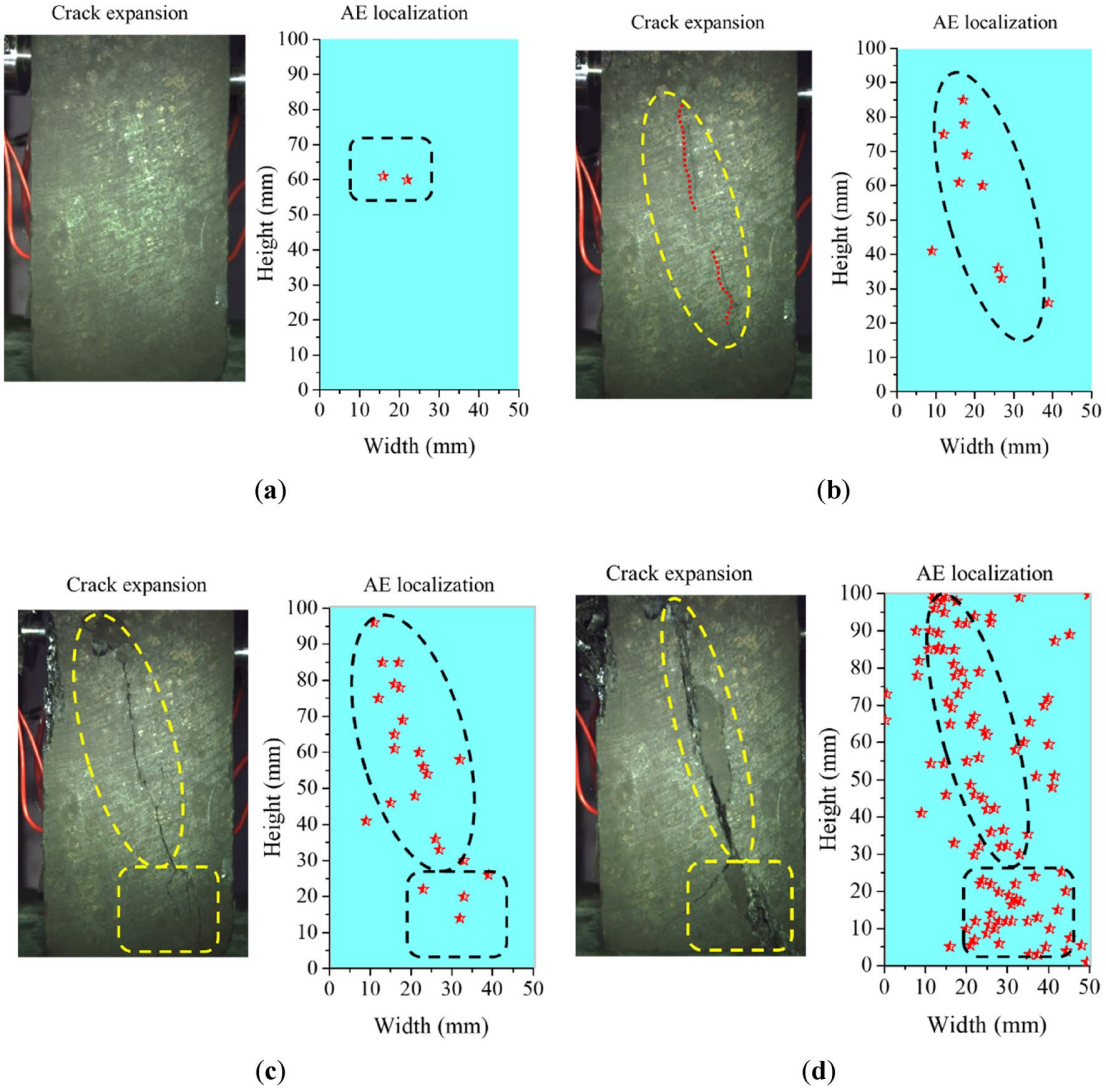
Results comparation between fracture events location and crack expansion before the failure of gas-bearing coal under load. (**a**) 100.1 s (37.1% maximum stress). (**b**) 299.1 s (80.3% maximum stress). (**c**) 359.6 s (93.7% maximum stress). (**d**) 396.9 s (100% maximum stress).

According to the moment tensor inversion method in “Moment tensor inversion method of AE signals” section, AE data collected at six monitoring points were used for moment tensor inversion. Therefore, obtaining location results of fracture events during loading process of gas-bearing coal were shown in Fig. 13. It can be seen that the red pentacle indicated the location of the fracture event. Due to the photographs of crack expansion were two-dimensional, the information of the front face of specimen was described. However, the results of moment tensor inversion were 3D. Therefore, it was necessary to project the 3D coordinate of the result to the front view for comparison as done in this study.

AE count records the number of waveform signals that the AE amplitude received by any sensors exceeds the threshold, that is, hit number. The number of AE events (fracture event) records the number of event that the six AE sensors all receive the same hit signal. Therefore, the number of AE events revealed by AE is much lower than that of AE count. As shown in Fig. 13, the fracture events of the specimen were few and cracks were insignificant in the early loading stage. In the later loading stage, fracture events significantly increased and cracks gradually expanded into a fracture zone and coalesced with each. The cracks on the specimen surface were concentrated, while the fracture events were distributed dispersedly. When the macrocrack zones and its influence zones were circled with yellow dotted line on the specimen surface, the fracture events in the zones were relatively concentrated correspondingly. Meanwhile, the connection directions of these zones concentrated with fracture events were basically consistent with the crack coalesence directions and their influence zone. In general, the location results of fracture events in the specimen were based on accurate calculation by AE moment tensor inversion method, which could reflect the distribution characteristics of damage localization evolution of coal specimen under loading stress and gas pressure.

### Statistical results of moment tensor inversion using AE signals

**Figure 14:**
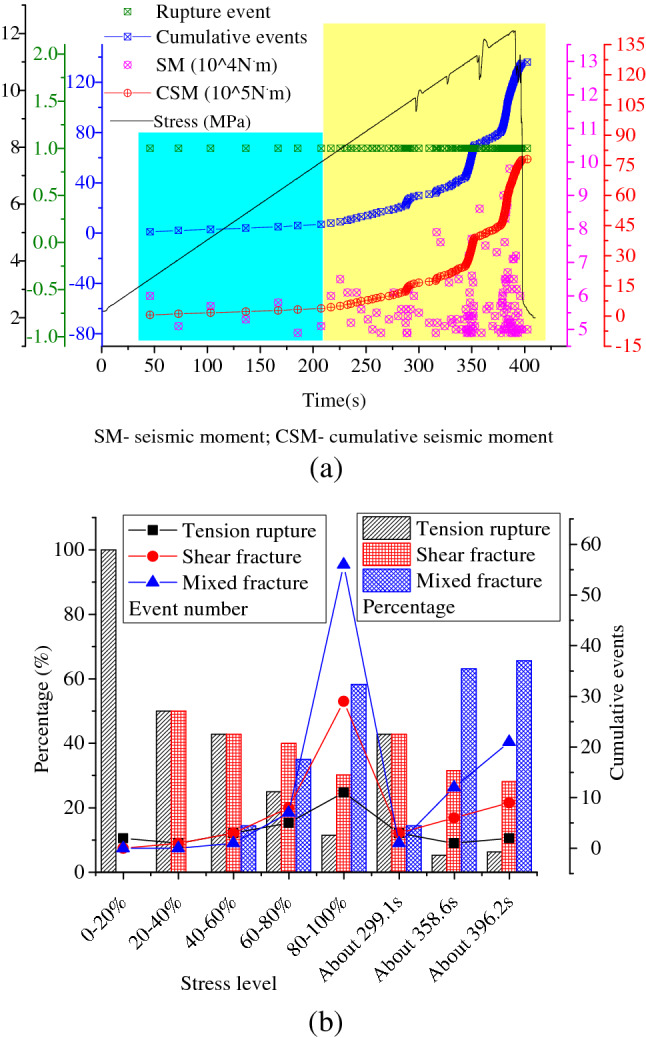
Statistical results of fracture events revealed by AE moment tensor inversion duirng the loading process of gas-bearing coal. (**a**) Temporal variation of fracture events with loading time. (**b**) Proportion of different types of fracture events.

The results of fracture events before the failure of gas-bearing coal under load were shown in Fig. 14. ① In early loading stage, the specimen belonged to compaction deformation and elastic deformation process. The micro-fracture activities were not active and the cumulative number of events was low. Almost all of fracture events were tensile fracture, and the average seismic moment values were low, which shows that primary cracks opened or closed under load. ② In the later loading stage, the specimen belonged to plastic deformation and damage process. The cumulative number of fracture events was high, while their occurring moments were relatively concentrated in time. With the increase of loading level, the increase speed of the event number was also accelerating. The seismic moment of fracture was dispersed significantly, but the overall value was higher, and the cumulative seismic moment increased rapidly. It reflected that the specimen damage was intensified and cracks initiated and expanded rapidly. Consequently, the elastic energy accumulated due to loading action was quickly dissipated. ③ As loading continues, the number and proportion of shear fracture events constantly rose with their influences enhanced. The number of mixed fracture events also increased, while the proportion decreased. Moreover, the proportion of tensile fracture reduced rapidly. At about 299.1, 359.6 and 396.9 s, the specimen was damaged seriously at local zones, resulting in sudden change of loading stress. And the cumulative count of fracture events and cumulative seismic moment rapidly increased at the above moments. Meanwhile, the proportion of shear fracture increased quickly and the average seismic moment was relatively high. Furthermore, significant crack expansion was found on the specimen surface at the above moments. At once, the degree of local damage in specimen was increased and the growth rate of fracture events was accelerated, and the proportion of shear fracture and average seismic moment rose. The intense zones and concentrated zones of fracture events were basically consistent with the concentrated zones of macrocrack and their influence zones, where the local coal mass was seriously damaged, regarded as the hazard zones to induce the dynamic failure of specimen. This indicates that tensile fracture played an important role in damage development and evolution during the crack initiation stage. When the specimen was locally damaged seriously, shear fracture was gradually dominant and played a crucial role in crack expansion and coalescence. ④ As the stress level approaches to peak, the specimen belonged to the critical failure state. Meanwhile, abundant microcracks gathered, coalesced and nucleated. It was accompanied by the decrease of local stress and extensive energy released. In addition, the AE count and energy suddenly rose and reached the peak (shown in Fig. 11). In this case, a large number of fracture events occurred and the average seismic moment rapidly rised. The cumulative seismic moment quickly increased to the peak and the fracture types of events were relatively stable, which was dominated by shear fracture. And the reason was that many new surfaces were formed due to rapid crack expansion and coalescence, at the same time shear slip and tear occurred on the fracture surface. Hence, AE count number was higher and a lot of shear fracture events occurred, while few microcracks simply opened or closed.

**Figure 15:**
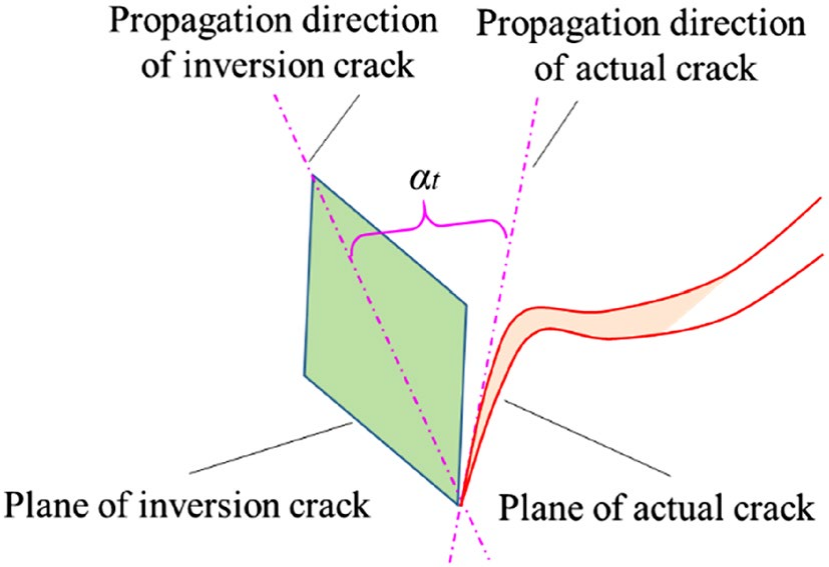
Schematic diagram of strike angle difference in crack expansion direction between AE moment tensor inversion results and actual measurement results.

**Figure 16:**
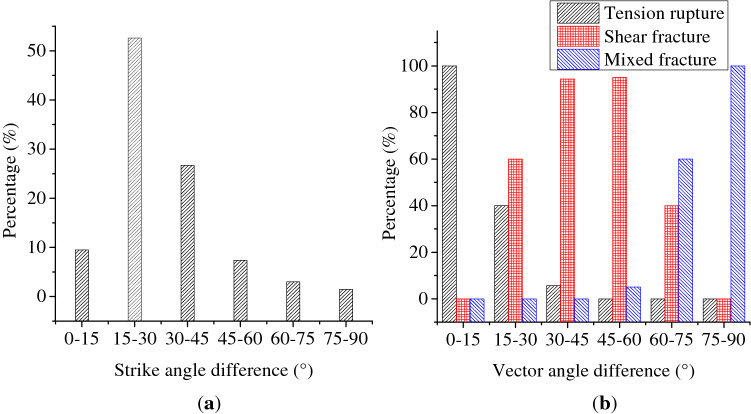
Statistics of fracture events during loading process of gas-bearing coal based on AE moment tensor inversion. (**a**) Difference in the stirke angle. (**b**) Difference in the vector angle.

The difference in the vector angle was defined as the angle between the displacement vector (direction of motion) of cracks and the normal vector of the crack surface obtained by the AE moment tensor inversion. As shown in Fig. 15, the difference in the strike angle was defined as the difference between the strike angle of crack surface on the front view (*x*–*z* plane in the Cartesian Coordinate System) obtained by AE inversion and the actual strike angle of cracks in the corresponding projection coordinates of the specimen on the front view. The smaller the difference in strike angle, the closer the crack expansion direction obtained through the AE inversion to the actual crack expansion direction. As shown in Fig. 16a, the differences in the vector angle and strike angle of all fracture events obtained by AE moment tensor inversion were summarized. The data of the difference between theoretical and actual strike angles of fracture events in the front view shows that the 88.9% of events had differences within 45° while the 62.2% within 30° (dominant in 15–30°). The camera could only record the cracks on the front face of specimen, while the AE moment tensor inversion results recorded the fracture events inside specimen with 3D characteristics. Therefore, considering the presence of systematical errors, the overall error of inversion results was still acceptable and reliable. As shown in Fig. 16b, the angles between the normal vector and motion vector of fracture surfaces from different fracture events were significantly different. The difference in the tensile fracture angle was mainly in the range of 0°–15°, while that of the shear fracture was mainly in 75°–90°. And the difference in the mixed fracture angle was distributed relatively in the range of 30°–60°. This indicated that the difference in the vector angles obtained through AE inversion in experimental results were basically consistent with theoretical analysis results. When coal mass was locally damaged violently, fracture events continuously occurred in a relatively concentrated zone. The fracture events were continuous in spread trend, and crack surfaces were coalesced with each in the development direction to form a stable crack zone, which was consistent with the photograph results of crack expansion recorded by camera. It showed that the AE moment tensor inversion method could locate the zones with violent local damage in gas-bearing coal specimen, evaluate the development trend of damage localization and identify the coalescence direction of crack zone inducing the final failure of coal specimen.

## Discussion

### Development of damage localization in gas-bearing coal

Coal mass is a kind of discontinuous medium, where the anisotropic structural planes with different sizes therein exist inside. Under the action of external stress, plastic deformation occurs in coal mass, and stress concentration preferentially appears at the tip of the primary crack. With the increase of the degree of stress concentration, cracks with weak joints and low strength in coal mass preferentially reach the rupture strength, which shows crack expansion and initiation of secondary cracks. However, not all cracks can continue to expand. Specifically, most of them will terminate, and only a few cracks will continue. Thus, the crack expansion mode shows bifurcation characteristics (the crack expansion mode has differences of “continuing to expansion” and “termination of expansion”).

It is because of the formation of local fracture failure zone, the continuous expansion and penetration of the fracture zone eventually induces the structural instability of the coal mass. The initiation and expansion of cracks are characterized by regionalization, which induces local damage aggravation and leads to overall structural damage. This phenomenon is called damage localization. The damage localization characteristics have multiple manifestations, such as localization, crack propagation localization, fracture event localization, et al. It can be regarded as the cause of damage and failure of gas-bearing coal, and also as the precursor of structural failure.

It is worth noting that after bifurcation characteristics appears, the crack expansion will be dense locally. Due to the “Matthew effect”, the advantage of crack expansion in the concentrated area will be strengthened and the regionalization characteristics are more significant. It shows that the belt characteristics and gradually forms local fracture failure zone. The continuous expansion and penetration of the fracture zone finally induced the failure of the coal structure. Therefore, the damage localization of gas-bearing coal is closely related to the state of crack propagation. The bifurcation of crack propagation mode can be regarded as the sign of damage localization.

### The influence of gas on AE response during gas-bearing coal loaded process

Compared with the loading process of coal mass without gas action, when the coal fully absorbs gas, both free gas and adsorbed gas exist inside the coal. On the one hand, free gas will apply vertical stress on the coal matrix surface and the crack surface, which can change the stress distribution. Thereby the values of critical strength will be changed for crack propagation and specimen failing. On the other hand, the adsorbed gas can "corrode" the structure of the coal matrix (as a non-mechanical effect), which reduces the cohesion between coal particles. Especially for the coal deformation and fracture, there will be a dynamic evolution of gas adsorption and desorption for coal, in the continuous development process of the closure of original crack and the generation and expansion of new cracks. Meanwhile, the free gas continuously impacts and loads the new fracture surface. Consequently, this will further "erode" the microstructure of the coal mass, which promotes the damage development. When the gas has compression, expansion and migration behaviors in a local confined space inside coal specimen, the gas pressure gradient changes dynamically, causing this effect to be more significant.

Generally speaking, for coal without gas action, the AE generated during the deformation and fracture process has a significant difference in various loading stages^14,34^. Normally, there are fewer AE signals in the early stage and more in the later stage. However, the frequent AE signals are more concentrated commonly. In contrast, the deformation and fracture process of gas-bearing coal has more significant non-linear characteristics. Affected by gas, the AE signals at different loading stages are more abundant. However, the elastic energy released by the coal mass is less when it fails, and the "absolute value" of AE signals are lower. It has been confirmed in the previous study results^35–38^. Therefore, based on the more abundant AE signals, the fracture event can be located more clearly and precisely, thereby better revealing the damage evolution process of coal mass. Moreover, the characteristics of fracture type are more obvious, whose reason is obvious. Under the action of gas, the "confining pressure" effect on coal mass under load is more significant, so that the shear characteristics are prominent. It also lays the foundation for the further utilization of AE signals to calculate the fracture types and reveal the precursor characteristics when coal specimen fails.

### Application of AE moment tensor inversion in gas-bearing coal

In essence, the damaging process on gas-bearing coal is that the internal primary defect and crack develop continuously under the action of stress concentration, which induces the damage localization evolution and final structure failure. In this development process of complex and nonlinear damage localization, AE signals can be used to spatially locate fracture events, analyze the properties and compositions of fracture, and obtain the spread trend of fracture surface.

For the deformation and fracture process of gas-bearing coal under loading stress as shown in the experimental results in “Experimental results” section, the moment tensor inversion results by using AE signals basically conform to the actual failure results of gas-bearing coal. The spatial distribution of fracture events, fracture types and spread direction of crack surfaces revealed by AE signals reflect the evolution process of damage localization and structural failure of coal mass. In particular, the increase of fracture events means that the damage degree is also improved. When the spread trend of fracture events is relatively continuous and basically consistent, it is conducive to the continuous expansion and coalescence of cracks. Finally, the gas-bearing coal is failed along the fracture zone. Meanwhile, crack initiation leads to the formation of new crack surfaces, accompanied by shear slip usually. The proportion of shear fracture events reveals the properties of crack expansion. When the specimen is seriously damaged locally, the shear fracture gradually plays a dominant role. With the continuous occurrence of shear expansion, the crack zone constantly changes from the ductile shear zone to the brittle shear zone. Meanwhile, the volumetric strain significantly increases, whichplays a key role in crack expansion and coalescence. The rapid increase of shear proportion at a high level can be regarded as precursor information of coal mass failure. At once, the average seismic moment also increases significantly.

Therefore, when monitoring the failure of gas-bearing coal under loading stress, the results of AE moment tensor inversion method can be utilized to identify the concentrated zones and fracture types of fracture events, and also to locate the fracture surface with serious local damage. Further, the orientation and direction of crack expansion and coalescence can be determined according to the strike of the crack surface of fracture events. Meanwhile, the data can be obtained based on the proportion of shear fracture events and the change of average seismic moment. As a result, the precursors can be acquired to forecast the final structural failure of coal mass, and the hazard zone of coal mass dynamic structural can be identified. It provides a practical study method for revealing the development process, occurrence area and spread direction of damage localization in gas-bearing coal.

## Conclusions

Based on the experimental study, the AE response characteristics and development process of damage localization were studied based on moment tensor inversion before the failure of coal mass under the coupling action of stress and gas. The conclusions were obtained as follows.

1. AE response characteristics were closely related to the damage degree of gas-bearing coal. As loading stress continues, the AE count and energy tended to rise. Moreover, AE signals became more abundant. When coal mass was severely damaged locally, the AE count and energy increased abruptly in a pulse pattern and reached the peak. During the deformation and fracture process of coal mass, cracks grew and propagated, meanwhile, elastic energy was released to the outside, regarded as the main cause of temporal response of AE signals.
2. Spatial distribution of fracture events in coal mass, meanwhile the motion vector and fracture intensity were obtained based on moment tensor inversion results by using AE signals. The concentrated zone and spread trend of fracture events in the inversion results were basically consistent with macrocrack coalesence direction and influence area. The differences basically conformed to theoretical analysis results in the strike angle and vector angle obtained through calculation, which could reveal damage localization evolution characteristics inside gas-bearing coal.
3. When fracture events were distributed concentratedly and spread continuously, cracks rapidly expanded and formed the macrocrack zone, and damage of coal mass was intensified. Before failure of coal mass, the proportion of shear fracture quickly increased and was at a high level. In this case, the average seismic moment significantly increased. Based on the moment tensor inversion results by using AE signals, the local damage area of coal mass could be identified and the strike of cracks could be predicted. Moreover, precursors can be obtained to forecast final failure of coal mass in the damage evolution, while the hazard zones of dynamic failure can be identified.

This study lays an experimental foundation for revealing the process of inoculation, evolution, and development of damaging localization in gas-bearing coal based on AE moment tensor inversion. Further, it provides a new idea for monitoring and forecasting dynamic disasters in deep coal mining further.

## Data Availability

All data, models, or code generated or used during the study are available from the corresponding author by request.
